# The Return to Nature; or, a Defence of the Vegetable Regimen: With Some Account of an Experiment Made during the Last Three Years in the Author's Family

**Published:** 1812-04

**Authors:** 


### CRITICAL ANALYSIS

### *OF RECENT PUBLICATIONS*

IN THE

### DIFFERENT BRANCHES OF PHYSIC, SURGERY, AND MEDICAL PHILOSOPHY.

*The Return to Nature; or, a Defence of the Vegetable Regimen: with some Account of an Experiment made during the last three Years in the Author's Family. By* JOHN FRANK NEWTON, esq. London. pp. 157.

MEN who take to the study of a science to which they were not originally educated, are commonly influenced by some decided turn of mind which generally imparts a character of novelty and originality to their observations, and perhaps constitutes what is commonly meant by the term genius. This observation is particularly illustrated by the medical profession, some of the greatest ornaments of which were originally destined for different walks of life. There are good reasons for believing that the celebrated Sydenham was in early life a soldier, and that he did not direct his attention to the means of saving the lives of his fellow-creatures, till the termination of the civil wars put an end to his occupation of destroying them. Mead, Boerhaave, and Buchan, were educated with the view of becoming calvanistic clergymen; and, *instar omnium*, the celebrated John Hunter, upon the fruits of whose labors, and the monuments of his genius, the present College of Surgeons may be said to be founded, in which men are now risen to eminence and wealth, whom he held in contempt for their ignorance, as they hated him for the the superiority of his genius, was bred to the humble occupation of a country carpenter!

In the department of dietetics, novelty of observation is particularly to be expected from those who may be termed interlopers in the profession, as the regularly bred are too much accustomed from their earliest years to put their faith in pills and potions, to be capable of devoting their attention to the operation of regimen; and, accordingly, we believe that more faith is put in the observations of Cornaro, and those of T. Wood, the famous miller of Billericay, as stated by Sir George Barker, than even in the doctrines of Cheyne, and other professed writers on the subject of diet.

We therefore regret that Mr. Newton has not candidly and simply detailed his own personal experience concerning the effects of regimen, unbiased by medical opinion or professional authority; instead of which he sets out, in the dedication to his book, with pinning his faith on Dr. Lambe, respecting the deleterious consequences of common, and the panacæal effects of distilled, water. Now, although we are far from denying the noxious effects of many kinds of water on the human body, used as drinks we cannot regard the experiments of Dr. Lambe, respecting the peculiar deleterious ingredients contained in common water, as conclusively correct; neither are we of opinion that the process of distillation is capable of depriving water of all its impurities, not even of all its metallic impregnations. And allowing all the importance of pure water to health, we should humbly apprehend that rain, or melted snow, which have undergone the distillation of nature, would afford the aqueous fluid at least in equal purity as that which had been subjected to the operation of culinary fire in metallic or even in glass vessels.

The use of distilled water, as a remedy for chronic disease, was recommended by the late excellent and judicious Dr. Heberden, (vide Transactions of the College of Physicians;) but he appears subsequently to have neglected it.

Mr. Newton commences with condemning the common use of too stimulant a diet for adults, and especially for children; a truth which, generally speaking, will not be disputed with him. But we can with equal truth aver, that we have seen very many examples of children affected with scrofula, who, after being kept upon a diet strictly vegetable, with the intention on the part of their parents to eradicate this constitutional tendency, have been decidedly injured; and, on the contrary, have recovered rapidly, when put upon a mixed diet of animal and vegetable food, combined with active exercise.

This work commences, as all treatises on vegetable regimen do, with an account of the prohibition of animal food to man, as first created, and the subsequent permission, not command, to eat it, when 'man had become carnivorous through sin.' In searching for the source of the many diseases that man is now the inheritor of, it is surprising that the author has overlooked the circumstance of the diet of man being twice altered, once at the fall, and again at the flood, without any correspondent change of his organisation being indicated to have taken place. Surely this affords a pregnant source of speculation to the mind occupied in such disquisitions!

The author next proceeds to introduce a variety of remarks respecting the deterioration of animal nature by the processes of civilisation and domestication. These observations we do not apprehend are justly applicable to human beings; for we conceive a state of society, and consequently of civilisation, to be the *natural* state of man.

As a specimen of the author's arguments in favor of a vegetable regimen, we select the following passage.

"Of all the children whom I have known or heard of, none has disliked fruit, but several have refused to eat meat. Some have been made sick with it. But it would rather be expected that distension and uneasiness would be the consequences of excess, and not sickness, of the stomach, where the food is of a quality entirely suited to the animal economy. This indication of what is or is not adapted to the human system, ought to have great weight; but I have still stronger ground to rest upon. It will not be denied that the race of men is, of all races, the most diseased; to such a degree, that the continuance of our lives, even in the usual instances of what is denominated longevity, by no means exceeds the period of maturity so much as among animals in general. Some individuals beyond a doubt have reached a great age. Not to mention Jenkins, old Thomas Parr of Shropshire, completed one hundred and fifty odd years, but cannot of course be supposed to have been sounder and healthier than the wild animals in their native woods. It therefore appears to follow, that if men were universally as healthy as these wild animals, they would as certainly exceed the age of one hundred and fifty years. As man reaches his full form and strength in his twenty-fifth year or thereabouts, it might be expected from the analogies of natural history that he would exist seven or eight times that period. This quickening of the step of death upon us, though it robs us of much of our own existence, is not unfavourable to that of the creation in general; for had it not been the heavenly dispensation that man, by living on animal food, should become unhearthy and rapidly perish, in the long progression of centuries he would have cleared the earth of all other animals; after which exhibition of his prowess, he might have had a more unanswerable plea than he at present has for making war on his own species. This insinuation must not be regarded as thrown out in the spirit of asperity; for here I would observe, once for all, that this essay is no vehicle of malignity and sarcasm. It would indeed be an ineffectual method of lowering our species, to trace their good qualities, as I am heartily disposed to do, to the nobleness of their nature, and their errors to the corruptions of society. Men are more to be commiserated than blamed for being driven by impulses, arising out of causes not sufficiently investigated, into the baseness of avarice, or the trammels of ambition. Many a headlong passion has been excited by the food and drink which have stimulated the brain through the stomach; and many an example of fatal despair has been exhibited to the world, from no other cause than that the channels of the secretions were clogged by the daily deglutition of substances ill adapted to the human constitution. Should a chemical analysis of the fluids ever be found within the reach of scientific ingenuity, to which Dr. Lambe's theory of constitutional diseases appears to point, it will then, and not till then, be explained how man, in quitting the nutriment on which alone nature had destined him to enjoy a state of perfect health, has debased his physical, and consequently his moral and intellectual, faculties, to a degree almost inconceivable. Real men have never been seen that we are aware of, nor has history, nor even poetry, depictured them. It is not man we have before us, but the wreck of man."

The author candidly warns those persons who may make the experiment of adopting the vegetable regimen, to expect no rapid change for the better, as two or three years at least must elapse before the crasis of the body can be changed, and the seeds of chronic disease eliminated. Mr. N. states the complaint from which he has been so far freed by the vegetable diet, as to be, in respect of his own feelings, quite a renovated being, to have been constitutional spasmodic asthma, doubtless one of the hardest knots that the wedges of materia medica have to separate.

He states, in a very decisive manner, p. 77, *that no ill effects have, in any instance, been felt from the adoption of this regimen.* This assertion is not supported by experience. We well know that dropsy and incurable palsy have, in many instances, been the result of too sudden a transition from a stimulant diet of animal food and wine, to a regimen of milk and vegetables.

The picture he draws of the condition of his own family under the influence of this mild diet, is pleasing and satisfactory.

"I may be mistaken, but I am persuaded that there is scarcely another instance in this never-ending metropolis of three grown persons, and four young children under nine years of age, incurring an expense of sixpence only for medicine and medical attendance in the course of two years; and that single charge was not made for either of the children, but for myself. This result is exactly what would be expected from the remarkably healthy appearance of the young people alluded to, which is so striking, that several medical men, who have seen and examined them with a scrutinizing eye, all agreed in the observation that they knew no where a whole family which equals them in robustness. Should the success of this experiment, now of three years' standing, proceed as it has begun, there is little doubt, I presume, that it must at length have some influence with the public, and that every parent who finds the illnesses of his family both afflicting and expensive, will say to himself, "Why should I any longer be imprudent and foolish enough to have my children sick?" All hail to the resolution which that sentence implies! But, until it becomes general, I feel it necessary to exhort in the warmest language I can think of, those who have young people in their charge, to institute an experiment which I have made before them with the completest success. To those domestic parents especially do I address myself, who, aware that temperance in enjoyment is the best warrant of its duration, feel how dangerous and how empty are all the feverous amusements of our assemblies, our dinners, and our theatres, compared with the genuine and tranquil pleasures of a happy little circle at home. Oh, if they knew the blessing of never hearing one's children restless at night to those who sleep in the midst of them; or of seeing one month, one year, of vigour, uniformly succeed another! The health of mine may be verified by the inspection of any strangers who shall be disposed to take that trouble. And surely it is to be presumed that their little ones also will be no less exempt from violent attacks after two or three years perseverance in a similar plan; that their forms will expand, their strength increase, in a very different ratio from the ordinary one: that the little family perturbations occasioned by the falls of children, which are in great measure attributable to the want of tone in their fibre, will be almost unknown; that as the fracture of limbs, like the rupture of blood-vessels, is more owing to the state of the body than to the violence of the shock encountered, they will be infinitely less liable to such distressing accidents; that their irritability, and consequently their objurgatory propensities, will gradually subside; that they will become not only more robust, but more beautiful; that their carriage will be erect, their step firm; that their development at a critical period of youth, the prematurity of which has been considered an evil, will be retarded: that, above all, the danger of being deprived of them will in every way diminish; while by these light repasts their hilarity will be augmented, and their intellects cleared, in a degree which shall astonishingly illustrate the delightful effects of this regimen."

The following is the bill of fare he presents to his readers. We by no means think it a well selected specimen of vegetable regimen, especially for children, and could suggest many many useful additions, as well as some salutary restrictions, were this the proper place or opportunity for so doing.

"It is not my intention to present the reader with a set of bills of fare for breakfast, dinner, and supper; but I will say a word or two of the manner in which we proceeded as to this particular. Our breakfast is composed of dried fruits, whether raisins, figs, or plums, with toasted bread or biscuits, and weak tea, always made of distilled water, with a moderate portion of milk in it. The children, who do not seem to like the flavor of tea, use milk and water instead of it. When butter is added to the toast, it is in a very small quantity. The dinner consists of potatoes, with some other vegetables, according as they happen to be in season; macaroni, a tart, or a pudding, with as few eggs in it as possible: to this is sometimes added a dessert. Onions, especially those from Portugal, may be stewed with a little walnut pickle and some other vegetable ingredients, for which no cook will be at a loss, so as to constitute an excellent sauce for all other vegetables. As to drinking, we are scarcely inclined on this cooling regimen to drink at all; but when it so happens, we take distilled water, having a still expressly for this purpose in our back-kitchen."

The author is disposed to attribute much of that habitual tendency of the blood, towards the brain, which he considers as the cause of a great part of that oddity of conduct that too frequently terminates in insanity, to the habitual effects of too stimulating a diet; and in this opinion we are disposed to concur with him.

The censures bestowed upon the medical profession are ill directed, and might well have been spared. They are introduced in the shape of a letter from a country cousin, but are evidently the production of the same pen. In sarcasm our author does not particularly shine; nor does ridicule indiscriminately appertain to a body of men honestly exercising a laborious and anxious profession, who, if the public did not find their services useful, would soon cease to exist. It is time enough to hold them up as objects of contempt, when the universal adoption of the vegetable regimen has rendered their exertions to counteract the consequences of luxury no longer necessary.

With this exception, Mr. Newton's work is composed in the style and manner of a scholar and a gentleman; and, whatever may be the ultimate determination respecting the most proper and salutary regimen for man in his present state of existence, we can truly state it as the opinion of one of the most eminent and enlightened medical practitioners of the present day, "that the efficacy of a vegetable and farinaceous regimen as means of preserving health, and of preventing or curing certain diseases, has not hitherto in this country had a fair trial." B.

*Practical*

